# Larger left hippocampal presubiculum is associated with lower risk of antisocial behavior in healthy adults with childhood conduct history

**DOI:** 10.1038/s41598-023-33198-9

**Published:** 2023-04-15

**Authors:** AmirHussein Abdolalizadeh, Kamyar Moradi, Mohammad Amin Dabbagh Ohadi, Fatemeh Sadat Mirfazeli, Reza Rajimehr

**Affiliations:** 1 Biological Psychology, Department of Psychology, School of Medicine and Health Sciences, Carl Von Ossietzky Universität Oldenburg, Oldenburg, Germany; 2 Interdisciplinary Neuroscience Research Program, Tehran University of Medical Sciences, Tehran, Iran; 3 Students’ Scientific Research Center, Tehran University of Medical Sciences, Tehran, Iran; 4 Mental Health Research Center, Psychosocial Health Research Institute, Department of Psychiatry, School of Medicine, Iran University of Medical Sciences, Tehran, Iran; 5 MRC Cognition and Brain Sciences Unit, University of Cambridge, Cambridge, UK

**Keywords:** Diseases of the nervous system, Psychiatric disorders

## Abstract

Conduct Disorder (CD) is defined as aggressive, antisocial, and rule-breaking behavior during childhood. It is a major risk factor for developing antisocial personality disorder (ASPD) in adulthood. However, nearly half the CDs do not develop ASPD. Identification of reversion factors seems crucial for proper interventions. We identified 40 subjects with childhood history of CD (CC) and 1166 control subjects (HC) from Human Connectome Project. Their psychiatric, emotional, impulsivity, and personality traits were extracted. An emotion recognition task-fMRI analysis was done. We also did subregion analysis of hippocampus and amygdala in 35 CC and 69 demographically matched HCs. CC subjects scored significantly higher in antisocial-related evaluations. No differences in task-fMRI activation of amygdala and hippocampus were observed. CCs had larger subfields of the left hippocampus: presubiculum, CA3, CA4, and dentate gyrus. Further, an interaction model revealed a significant presubiculum volume × group association with antisocial, aggression, and agreeableness scores. Our study shows that healthy young adults with a prior history of CD still exhibit some forms of antisocial-like behavior with larger left hippocampal subfields, including presubiculum that also explains the variability in antisocial behavior. These larger left hippocampal subfield volumes may play a protective role against CD to ASPD conversion.

## Introduction

Conduct disorder (CD) is a serious neurodevelopmental disorder defined by a persistent pattern of violence and flouting rules and social norms that significantly impact families and society^1,2^. It affects school-aged children and is more prevalent in boys. Its etiology is complex with various genetic (mainly affecting serotonergic and dopaminergic neurotransmission) and environmental risk factors (e.g., maternal smoking and alcohol consumption, malnutrition)^3^. CD may accompany many psycho-developmental issues such as substance abuse, depression, anger problems, and in some instances, the lack of empathy, which is the hallmark of callous-unemotional (CU) traits. CD is also a major risk factor for developing antisocial personality disorder (ASPD)^2,4^. The behavioral hallmarks of the ASPD are the same as CD, and it also follows the same gender difference: it is more prevalent in males. Although it’s prevalence is approximately 2–3% in general population, it has been found in prisons to a higher degree as 47% in male inmates^5^.

Similar patterns of functional or structural disturbances have been seen in both CD and ASPD. For example, functional MRI (fMRI) studies in patients with ASPD suggest significant deficits in the networks related to social cognition and responses to threats^6^. In line with this finding, reduced amygdala response in tasks involving emotions has also been seen in CD^7^. A recent functional MRI meta-analysis also revealed a constant brain activation decrease in right basolateral amygdala, the dorsomedial prefrontal cortex, and lateral prefrontal cortex in psychopathy^8^. The basolateral nuclei of amygdala is involved in anxiety, fear conditioning, and social network size and complexity. Its connections to the hippocampal CA1 regulates anxiety, social behavior, and emotion-modulated spatial memory^9^, the behaviors that are generally disturbed in both CD and ASPD^10,11^. Structural MRI studies also supported a lack of social cognition and emotion in CD subjects. There is evidence of decreased gray matter in various parts of the brain associated with such responses, including lower volumes of the left amygdala, right insula, left medial superior frontal gyrus, and left fusiform gyrus in CD patients^4,12^. On the other hand, some studies observed increased gray matter of the orbitofrontal cortex while others reported reduced volume comparing CD adults with healthy subjects^13,14^. Also, voxel-based analysis of patients with ASPD revealed grey matter reduction in the left frontopolar cortex and the paralimbic region associated with memory and emotional problems in these subjects^15,16^. Smaller left hippocampus and amygdala are observed in CD patients^17^. However, in another study, no differences in amygdala volume and a smaller right hippocampus is noted in CD with behavioral correlates^18^. This discrepancy in results may be due to using different imaging protocols, analytical pipelines, or looking at the structures as a whole, rather than looking directly into substructures.

Structural and functional changes in the orbitofrontal-paralimbic system and hippocampal-amygdala complex both in CD and ASPD suggest them as a possible prognostic neural marker for children with CD for developing into ASPD in the future^17,19^. However, there is a lack of research regarding neural changes and socio-emotional profiles in healthy young adults with a history of CD in their childhood. In one of the few studies in this regard, women with prior history of CD have reduced grey matter volume in the hippocampus, associated with antisocial behavior^20^.

Therefore, we aimed to investigate the psychiatric and socio-emotional aspects of current healthy adults who had a prior history of CD in their childhood, detecting any possible deviation from normal psychiatric or emotional states. Moreover, we aimed to identify neural correlates of childhood CD by evaluating whole-brain volumetric measures, hippocampal subfields, and amygdala subnuclei, their activity during an emotional task-fMRI, and their respective associations with psychiatric and emotional evaluations. We are specifically interested in hippocampus and amygdala structures due to their possible involvement in CD and ASPD psychopathology based on the literature. Studying the substructures using a high-resolution imaging and a hippocampoamygdalar-directed analytic pipeline helps us to first, overcome the discrepancy in results due to analytical approaches, and second, better delineate the possible psychopathology based on the substructures involved. These measurements may lead us to identify the neural underpinnings of the different neurodevelopmental pathway which encourages the conversion of CD into healthy adults rather than developing ASPD.

## Materials and methods

### Participants

We used the Human Connectome Project young adults (HCP-YA) for the current study^21^. It includes 1206 young adults from 25 to 36 years old who have no current psychiatric or neurologic disorders. They have acquired high-resolution structural, diffusion-weighted, and functional MRI data, plus several neuropsychological and cognitive tests and tasks. We identified 40 subjects who had a diagnosis of conduct disorder in their childhood (CC) based on the DSM-5 (The Diagnostic and Statistical Manual of Mental Disorders) conduct disorder criteria extracted from Semi-Structured Assessment for the Genetics of Alcoholism (SSAGA) questionnaires^22^ answered by the participants. The antisocial history part of the SSAGA obtained by HCP includes 24 questions regarding the presence of conduct-, or antisocial-like behavioral patterns during childhood and adolescence, their prevalence, duration, and starting date. We identified questions corresponding to the DSM-5’s conduct disorder criteria and their duration. The subjects scoring the minimum three out of fifteen criteria, with at least one symptom present for more than six months were considered to have conduct disorder history in their childhood (Supplementary Table 1). We included subjects who have at least three symptoms happening within a 6-months period. The remaining subjects who did not have history of conduct disorder in their childhood were considered as the control group (n = 1166; HC).

All procedures in subject recruitment and data sharing were approved by Washington University Institutional Review Board (IRB)^23^. The subjects were recruited after signing an informed consent. We accessed the de-identified data after accepting open and restricted data access agreements of the HCP. Moreover, our data analysis was performed in accordance with ethical guidelines of the Iran University of Medical Sciences, Tehran, Iran.

### Evaluations

#### Demographics

Subject demographics included age, sex, and years of education provided by HCP. We included total household income level as an indicator of socioeconomic status of the subjects, categorized into eight levels from one to eight: 1 = less than 10,000$, 2 = 10,000 to 19,999$, 3 = 20,000 to 29,999$, 4 = 30,000 to 39,999$, 5 = 40,000 to 49,999$, 6 = 50,000 to 74,999$, 7 = 75,000 to 99,999$, and 8 = 100,000$ and more. Penn Progressive Matrices (PMAT) was also included as a measure of fluid intelligence^24^.

#### Emotional

We used the NIH Toolbox Emotion Battery (NIHTB-EB)^25^ and Penn Emotion Recognition Task (ER40)^26^ for evaluating the emotional aspects of the subjects. The NIHTB-EB includes a variety of tests evaluating negative affect (sadness, fear, anger), psychological well-being (positive affect, life satisfaction, meaning and purpose), social relationships (social support, companionship, social distress, positive social development), and stress and self-efficacy (perceived stress, self-efficacy). In the ER40, each subject is asked about the emotion of a set of 40 faces in five categories: angry, sad, happy, fear, and neutral. The number of correct responses and median response time for the right answers is measured.

#### Psychiatric and life function

We used scores based on Achenbach Adult Self Report (ASR)^27^ to evaluate the psychiatric and life function of the subjects. ASR has two components: the syndrome scale and the DSM-oriented scale. We included the anxious/depressed scale, withdrawn, somatic complaints, thought problems, attention problems, aggressive behavior, rule-breaking behavior, intrusiveness, internalizing and externalizing behaviors, and total ASR scores from the syndrome scale. From the DSM-oriented scale, we included DSM depressive, anxiety, somatic, avoidant personality, attention-deficit/hyperactivity, and antisocial personality problems. We included all the ASR-based scores of the subjects in the current study as sex and age-adjusted T-scores.

#### Personality

Personality traits of subjects were acquired based on NEO five-factor inventory (NEO-FFI)^28^. This 60-item questionnaire can be used to study the personality construct of an individual to “agreeableness,” “openness,” “neuroticism,” “extraversion,” and “conscientiousness”.

#### Impulsivity

We used delay discounting task as a measure of impulsivity^29^. In this task, the subjects are given the choice of a reward now or a more valuable reward with a delay. The trials are repeated with changes following a set of rules until an *indifference point* is achieved: the subjective value of the later more valuable reward is equal to the present less valuable reward. Plotting the indifference point reward values against delay time (both normalized) results in a hyperbolic graph. The area under the curve (AUC) of this graph is considered as a measure of impulsivity: higher impulsivity results in smaller present reward values preferred (a.k.a., considered being equal) to a shorter delay interval, resulting in a steeper hyperbolic chart and lesser AUC^30^. We included delay discounting AUCs of trials with 200$ and 40,000$ from HCP.

### Imaging

#### Acquisition and preprocessing of structural data

High-resolution T1 and T2 scans were acquired using 3 T Connectome Siemens Skyra with the following parameters: T1: 3D-MPRAGE, TR = 2400 ms, TE = 2.14 ms, TI = 1000 ms, FOV = 224 × 224, Voxelsize = 0.7 mm isotropic; T2: T2-SPACE, TR = 3200 ms, TE = 565 ms, FOV = 224 × 224, Voxelsize = 0.7 mm isotropic. We used the minimally preprocessed structural data, available as structural extended preprocessed^31^. The analysis pipeline, which resulted in the minimal preprocessed structural data, is briefly as follows: All structural acquisitions were first corrected for gradient distortion, aligned and averaged, skull stripped, corrected for readout distortion, and then fed to the *recon-all* pipeline of FreeSurfer^32^, using both high-resolution T1 and T2 weighted images for better identification of pial surfaces. Finally, morphometric measures of cortical parcellations and subcortical segmentations were calculated based on the Deskian-Killiany atlas^33^. The preprocessed data were available for 35 CC subjects. We matched these subjects with 69 HCs based on age, and education using *matchIt* library in R^34^.

#### Hippocampal/Amygdala segmentation

**Figure 1:**
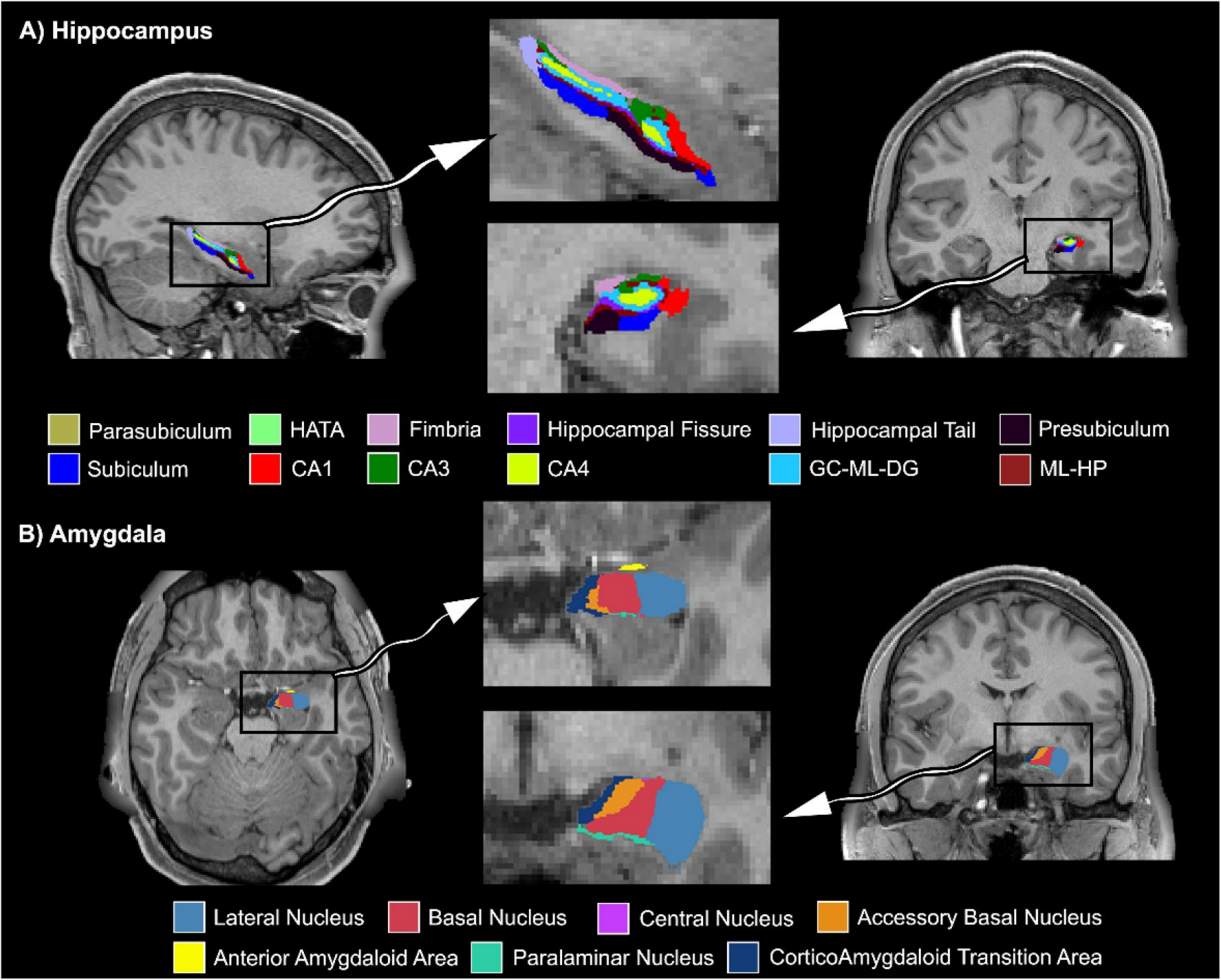
Left hippocampal subfields (**A**) and subnuclei of amygdala (**B**) in one of the subjects labeled on the T1 MRI scan. HATA: Hippocampo-amygdalar transition area, CA: Cornu Ammonis, GC-ML-DG: Granule cell and molecular layer of dentate gyrus, ML-HP: Molecular layer of hippocampus.

We used segmentation of hippocampal subfields and subnuclei of the amygdala implemented in FreeSurfer v7.1.1^35,36^. This automated approach provides the hippocampus and amygdala segmentation based on a high-resolution probabilistic atlas derived from fifteen autopsy samples. We used high-resolution (starting with hires_) T1 and T2 scans provided in the FreeSurfer output */mri* folder in structural extended preprocessed data by renaming the associated files as an input for *segmentHA_T2.sh* command (Fig. 1). The analysis was run on the 35 CC and 69 HC subjects using the *parallel* command in Linux Ubuntu 18.04 to use all the CPU threads^37^. Divisions of segments were summed to include that segment as a single volume (e.g., CA1 equals CA1 head plus CA1 body).

#### Emotion processing task fMRI

We chose this paradigm to evaluate differences in emotion processing between CC and HC, considering the differences observed in the previous studies between subjects with CD or ASPD. The whole-brain EPI acquisition done by the same 3 T Siemens Skyra scanner parameters were as follows: 32 channel head coil, TR = 720 ms, TE = 33.1 ms, flip angle = 52 deg, BW = 2290 Hz/Px, FOV = 208 × 180 mm, 2.0 mm isotropic voxelsize, with a multi-band acceleration factor of 8. Two task fMRI runs were done, one with right-to-left and another with left-to-right phase encoding directions. The procedure of task fMRI is described elsewhere^38^. The emotion task fMRI is adapted from Hariri et al.^39^, in which the subjects were asked to match the two shown pictures of faces or shapes on the lower part of the screen to the one presented above. The faces had either fearful or angry expressions and the shapes were depicting fearful or threatening situations. It was shown by Hariri et al., that face vs. shapes contrast shows a reliably strong activation of the bilateral amygdala indicating selective activation of amygdala to negative facial stimuli. Task fMRI data was minimally preprocessed and made available by HCP^31^. In brief, the data were gradient distortion corrected, motion-corrected, EPI distortion corrected, and then registered non-linearly with the “surface-based” method to MNI registered subject’s T1 scans. The minimally preprocessed data was then fed to FSL FEAT to apply contrasts and derive betas ^40^. We used the faces vs. shapes contrast grayordinates stats (cope3.feat/cope1.dtseries.nii) analyzed with 2 mm FWMH (full-width at half maximum) smoothed task fMRI data of 1039 subjects (36 CC and 1003 HC) provided by HCP. Mean beta values of bilateral amygdala and hippocampi were extracted using cifti-matlab commands (https://github.com/Washington-University/cifti-matlab). CIFTI-indices associated for each region is as follows: left amygdala = 59,688 to 60,002, right amygdala = 60,003 to 60,334, left hippocampus = 84,561 to 85,324, and right hippocampus = 85,325 to 86,119.

### Statistical analysis

We used Rstudio software based on R statistical package version 3.6.1^41,42^. Subject demographics and evaluations were compared between 40 CC and the rest of the HCP subjects (HC; n = 1166), using chi-square, *t* test, or Mann–Whitney test based on the type and distribution of the data.

We used clusterwise correction for multiple comparisons (*mri_glmfit-sim* command in FreeSurfer) with 5000 permutations, vertex-wise *p* value < 0.001 and cluster-wise *p* < 0.05 covarying age and gender to identify between group differences in cortical parcellation thickness^43^. To evaluate structural volumetric differences between two groups, differences between FreeSurfer generated subcortical volumes were evaluated and were adjusted for age, sex, and total intracranial volume. The same model was also applied for hippocampal and amygdala segmentation volumes with the addition of education and subject income level to the covariates. Next, we decided to evaluate how the differences in behavioral evaluations (part 2.2) can be described by the different structural volumetry seen in the segmentation analysis. Thus, we used a GLM using the former as the outcome and the interaction between significantly different segmentation volumes and group variable (hippocampal or amygdala × group) as the predictor variable with the same covariates as the previous model in the age-, and education-matched subsample of the subjects (35 CC, 69 HC). To overcome the multiple testing problem, we used the Benjamini-Hochberg’s False Discovery Rate (FDR) method to correct the *p* values wherever applicable^44^. Corrected *p* values less than 0.05 were considered to be statistically significant.

## Results

### Demographics and evaluations

After correcting for multiple comparisons and compared to HCs, CC subjects had higher male percentage, anger affect, anger aggression, antisocial personality and attention-deficit/hyperactivity problems, aggressive and rule-breaking behavior, intrusiveness, thought problems, externalizing, and total ASR scores. They also had lower number of correct neutral emotion identification (part of ER40) and agreeableness compared to HCs. Significant differences are shown in Table 1. All between group comparisons of demographics and evaluations can be seen in supplementary Table 2.

**Table 1:** Significant demographics and evaluations differences between two groups.

Variables	CC	HC	Corrected *p*
	n = 1166	n = 40	
Demographics			
Sex (M (%))	521 (44.7)	29 (72.5)	0.008*
Emotion			
Penn emotion recognition test			
Number of correct neutral identifications	7.13 (1.25)	6.53 (1.71)	0.037*
NIH toolbox: emotion battery			
Anger affect	47.90 (8.28)	51.80 (10.92)	0.040*
Anger aggression	51.88 (8.69)	60.47 (10.48)	< 0.001*
Personality			
Agreeableness	33.44 (5.77)	30.00 (6.46)	0.008*
Psychiatry and life function			
DSM-oriented ASR (T scores)			
Antisocial personality problems	52.94 (4.53)	57.73 (9.79)	< 0.001*
Attention-Deficit/Hyperactivity Problems	54.79 (5.62)	58.25 (9.40)	0.040*
Syndrome scale of ASR			
Aggressive Behavior	52.60 (3.90)	56.23 (9.31)	0.015*
Rule breaking behavior	53.80 (5.01)	58.62 (9.36)	0.001*
Intrusive	53.75 (5.44)	56.98 (7.40)	0.012*
Thought problems	53.70 (5.68)	56.17 (7.67)	0.040*
Externalizing	48.70 (8.76)	55.85 (12.20)	0.001*
ASR total score	47.90 (8.84)	53.05 (12.04)	0.016*

*HC* healthy controls with no history of conduct, *CC* childhood conduct history, *M* male, *DSM* the diagnostic and statistical manual of mental disorders, *ASR* adult self-report.

We investigated between-group differences after randomly matching HCs with CCs. Most of the differences above remained significant; the significant differences for thought problems and anger affect disappeared. Also, in this subsample, CCs showed higher attention problems (Supplementary table 3).

### Imaging

#### Whole-brain volumetric study

No clusters of cortical vertices show different thickness between groups (max. -log(*p*) of a significant vertex: left hemisphere = 3.27, right hemisphere = 2.84). There were no significant differences between CC and HC after Benjamini–Hochberg correction in subcortical volumes (Supplementary Table 4).

#### Hippocampal subfields and amygdala subnuclei

**Figure 2:**
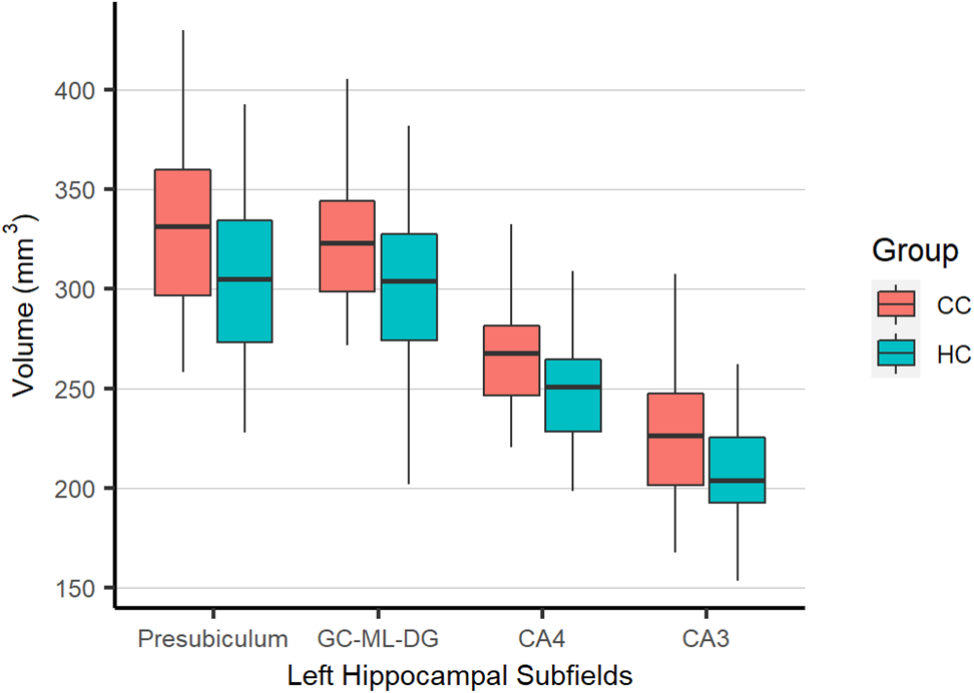
A boxplot showing significant left hippocampal subfield differences between two groups (all corrected *p* values < 0.05). CC: Childhood history of Conduct, HC: Healthy Controls, GC-ML-DG: Granule cell and molecular layer of dentate gyrus, CA: Cornu Ammonis.

CC subjects had larger CA3, CA4, presubiculum, GC-ML (Granule cell and molecular layer) of the dentate gyrus, in the left side compared to the controls using a statistical model adjusted for age, sex, education, income, and total intracranial volume (Fig. 2). Left hippocampoamygdalar transition area (HATA) was also larger in CC compared to HC (mm^3^; mean (SD): 68.38 (10.07) versus 61.52 (9.23), corrected *p* = 0.036). There were no differences between amygdala subnuclei or right-side hippocampal subfields between two groups (Supplementary Table 5).

#### Emotion task fMRI of hippocampus and amygdala

Faces versus shapes contrast beta values of emotion task fMRI was available for 1039 subjects (36 CC and 1003 HC). Using an ANCOVA model and adjusting for age, sex, education, and income level, there were no significant differences between mean beta values of the two groups in the left (HC versus CC (mean (SD)): 30.22 (18.12) versus 32.77 (18.60), F (1, 1026) = 0.64, *p* = 0.42) and right (HC versus CC (mean (SD)): 31.55 (18.57) versus 29.73 (17.21), F (1, 1026) = 0.27, *p* = 0.60) amygdala. There were also no significant differences between mean beta values of the two groups in the left (HC versus CC (mean (SD)): 8.69 (14.67) versus 11.12 (14.07), F (1, 1026) = 0.68, *p* = 0.40) and right (HC versus CC (mean (SD)): 9.95 (13.04) versus 10.69 (14.46), F (1, 1026) = 0.09, *p* = 0.75) hippocampus.

#### Association of evaluations with hippocampal subfields and amygdala nuclei volumes

**Figure 3:**
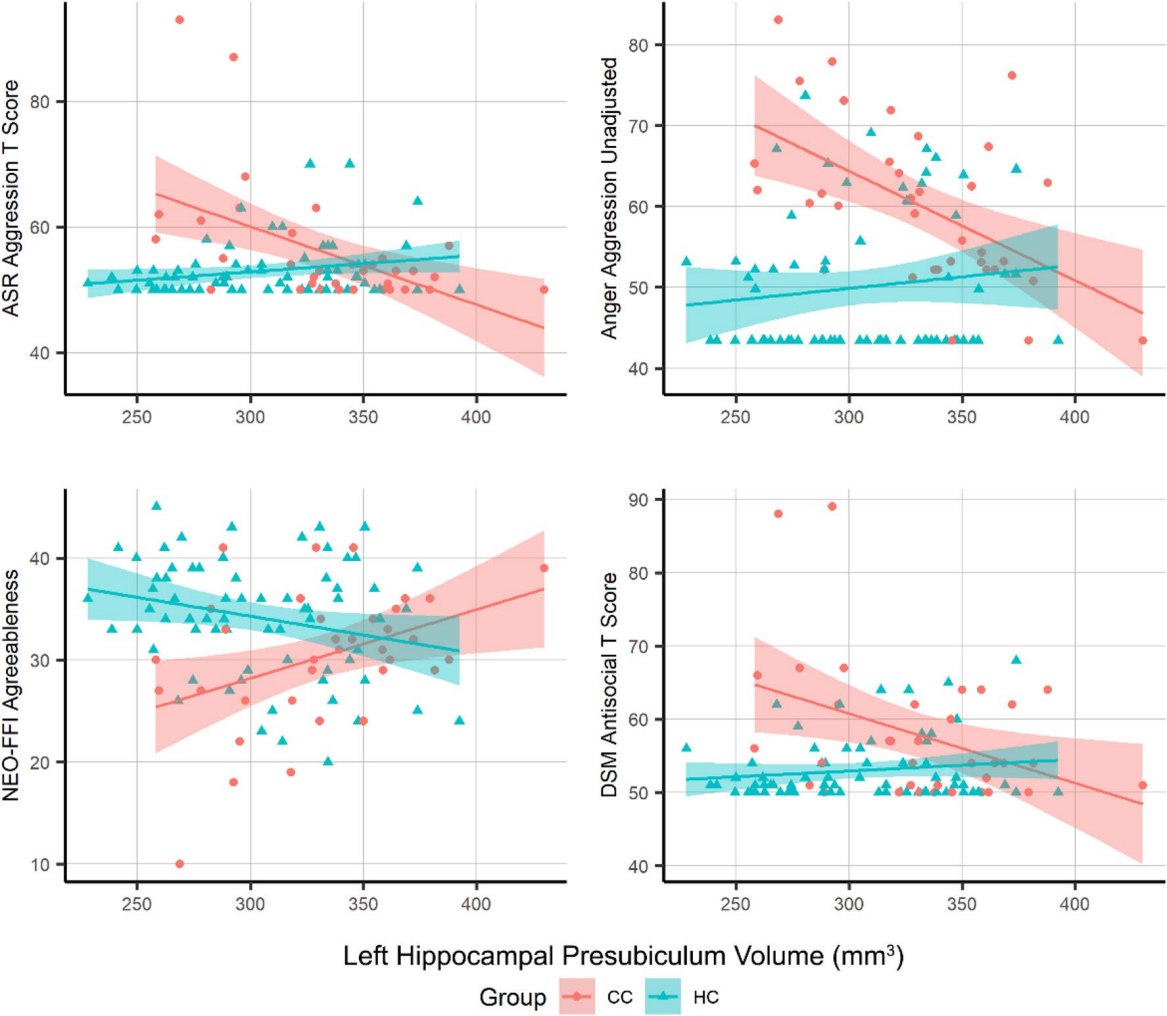
Significant interaction model results that were between left hippocampal presubiculum and four behavioral variables. CC: Childhood history of Conduct, HC: Healthy Controls, ASR: Adult Self Report.

We then entered the significant between-group difference results of evaluations and hippocampal subfields into an interaction analysis. We were specifically interested in the extent of behavioral differences that may be explained by the differences in the subfield volumes. After correcting the interaction model results for multiple comparisons, the models were significant for left hippocampal presubiculum and DSM antisocial T score, ASR aggression T score, anger aggression, and agreeableness from personality assessments (all corrected *p* < 0.05; Fig. 3, supplementary Table 6).

## Discussion

In the present study, we provided novel evidence regarding the effect of the previous history of CD in childhood on the behavioral, emotional, and neurobiological features of currently healthy adults. The behavioral parameters were systematically compared between CC and HC groups in four major domains: emotion, psychiatric and life functions, impulsivity, and personality. Significant differences were reported as a higher male percentage, anger affect, anger aggression, antisocial personality, and attention-deficit/hyperactivity problems, aggressive and rule-breaking behavior, intrusiveness, thought problems, externalizing, and total ASR scores, and lower agreeableness and neutral emotion recognition in the CC group. From a neuroimaging perspective, the cortical thickness and volume of none of the brain regions or amygdala nuclei were different between the two groups. However, the CC group had larger CA3, CA4, presubiculum, GC-ML, and HATA in the left hippocampus. Interestingly, left hippocampal presubiculum volume was significantly associated with DSM antisocial T score, ASR aggression T score, anger aggression, and agreeableness using a group × volume interaction model; larger left presubiculum volumes were associated with lower aggression and antisocial personality, and higher agreeableness in CCs, but not in HCs.

There is robust evidence regarding the significant association between child- and adolescent-onset CD and adulthood antisocial personality disorder^45^. However, childhood CD does not necessarily progress to antisocial disorder as a proportion of subjects with CD experience recovery, especially those with low psychopathic traits^46^. The improvement from CD to the normal condition has been attributed to various biological and environmental factors such as less initial severity of CD, higher child verbal intelligence, greater family socioeconomic status, and not having antisocial biological parents^47^. While this is probable for children with CD to improve to nearly normal and healthy status in case of receiving appropriate cares and treatments, surprisingly, to the best of our knowledge, few studies have ever investigated the behavioral and neurobiological outcomes of this population in adulthood, and this study is considered among the first of its kind in this topic. Our findings suggest that although subjects with CD-to-normal status reversion do not meet the criteria for adult-onset antisocial personality disorder, they still show some antisocial traits. Most of these traits are also evident in antisocial personality disorder (e.g., rule-breaking, aggressive behavior), and the mere difference might be in the trait of the symptoms.

Callous-unemotional (CU) traits are a personality-related subgroup of psychopathic personality traits, characterized by low empathy, restricted affect, interpersonal callousness, and lack of motivation and are significantly associated with more severe and more persistent antisocial behaviors^48^. Although subjects with the previous CD still exhibited some antisocial traits, the emotion and personality domains were found within an almost normal range, confirming the low levels of CU traits in our participants which help their improvement over time in antisocial behaviors.

Literature has addressed alterations in brain structure or structural connectivity of youths with CD, oppositional defiant disorder (ODD), and conduct problems^3^. Particularly, according to a meta-analysis of six studies, youths with child-onset conduct problems showed decreased grey matter volume in the left amygdala and anterior insula compared to typically developing ones^12^. Notably, all previous studies have investigated the alterations in brain structures in patients with current conduct problems. For the first time, this study showed that adults with prior CC and current healthy status exhibit larger subfields in the left hippocampus (CA3, CA4, Presubiculum, GC-ML of DG, and HATA) compared to individuals with no history of conduct problems during their lifetime. It was suggested that children with CD have smaller hippocampus compared to matched healthy controls, which was found associated with higher impulsive behaviors^17^. Childhood maltreatment, as a major risk factor for developing CD, is associated with smaller CA1, CA3, presubiculum, and DG on the left side^49^. In another study, Dahmen et al., replicated smaller CA1, CA3, and DG in the subjects with early life adversity^50^. On the other hand, our CC group consists of participants with a history of CD that have experienced behavioral recovery. Based on the association between smaller subfields of the left hippocampus and CD, the larger hippocampus in the CC group might be justified by the hypothesis that the larger hippocampus has a protective role in CD and increases the chance of recovery. The protective role of the hippocampus volume has been mentioned in a number of previous studies^51,52^.

We showed that in addition to the enlarged left hippocampus subfields in the normal participants with CC, there is a negative association between presubiculum volume and antisocial-like traits observed in our population. The behavioral and clinical correlates of the presubiculum are not clearly understood. Clinical studies have shown its involvement in the early stages of Alzheimer’s disease^53,54^ and schizophrenia^55,56^. In a relevant finding to our study, left presubiculum volume was negatively correlated with the severity of childhood maltreatment ^57^ which is a major contributor to the emergence of conduct disorder^58^. On the other hand, behavioral studies pinpoint its activation in viewing scenery^59^, and that it positions cells involved in the head-direction system^60,61^. Older studies in animal models showed that it is involved in connecting the hippocampus to anterior thalamic nuclei^62,63^. Interestingly, in a study using tracers, it was shown that the dorsolateral prefrontal cortex (DLPFC) is connected to the presubiculum via two pathways: the medial pathway traverses through the cingulum bundle, and the lateral pathway via the fronto-occipital pathway^64^. Altered DLPFC structure and function is a common finding in antisocial behavior, esp. on the left side^65^. Lesions to DLPFC, either due to trauma^66^ or artificially induced due to inhibitory transcranial magnetic stimulation^67^, result in a higher tendency to aggressive behavior in the affected subjects. The effects are more pronounced in the left side DLPFC inhibition. Our study is the first to show that the left hippocampal presubiculum may contribute to antisocial and aggressive behavior, and probably its connections with left DLPFC may be an important factor.

One interesting finding in our study was a left-sided hippocampal laterality considering the neurodevelopmental differences in hippocampus^68^ and the neurodevelopmental nature of the conduct disorder^2^. Previous studies on the hippocampal asymmetry in violent offenders and psychopathy has concluded a larger right-sided hippocampal volume^69^, and lower activity in the left side In a positron-emission-tomography study^70^. Accordingly, Soderstrom et al.^71^ showed an association between reduced left hippocampal activaty and high psychopathy scores in violent offenders. This association was not observed in right hippocampal activity. These findings are all consistent with our study on the protective role of left hippocampus against antisocial behavior.

Numerous studies have addressed the association of aggressive behaviors with amygdala function. Patients with refractory aggressive behaviors have benefited from amygdala surgeries^72^. Amygdala is considered the key region for regulating aggressive tendencies^73,74^. It has been shown that aggressive behaviors are correlated with the size of the amygdala. A negative association was reported between amygdala size and aggressiveness in healthy individuals^75^. On the other hand, several studies have suggested that CD patients have lower amygdala size as well as higher aggressive tendencies. A few studies have attributed the CD-induced aggression to changes in amygdala structure and function^76,77^. Despite these studies, our study showed no differences in amygdala volumes nor their activity in a emotion-recognition task fMRI between two groups.

Several limitations should be considered. First, the limited sample size restricted the generalizability of the results. Second, the cross-sectional setting of this study prevents us from understanding the specific disease course over the subjects’ lifetimes. Although we have used the high-resolution structural MRI data of the human connectome project and latest FreeSurfer hippocampal and amygdala segmentation pipeline, it has to be advised that using neuroimaging to study subnuclei that are small and hard to define may not be an ideal choice. Furthermore, diagnosis of CD has been done retrospectively in this study. Studying the children with CD in a prospective cohort design could overcome this limitation.

In conclusion, we, for the first time, evaluated the behavioral, emotional, and neurobiological performance of healthy adults with a previous history of CC. Examining the brain structure in this population, we found enlarged left hippocampal subfields and a negative association between the volume of presubiculum of left hippocampus and antisocial-traits in people who have recovered from childhood conduct disorder. This study suggests that CC does not necessarily progress to antisocial personality disorder; however, some antisocial traits remain in adulthood and left presubiculum volume may play a protective role against conduct to antisocial conversion. Further studies are required to confirm our preliminary results.

## Supplementary Information

Supplementary Information.

## Data Availability

Raw data of this study is available via Human Connectome Project (https://db.humanconnectome.org). The analyzed data and R scripts used for the statistical analysis (including statistical figures) of this study are available upon request from corresponding author or the first author.
