# Supplementary material for: Larger left hippocampal presubiculum is associated with lower risk of antisocial behavior in healthy adults with childhood conduct history
Source: Sci Rep. 2023 Apr 15;13:6148. doi: 10.1038/s41598-023-33198-9 (PMC10105780; doi:10.1038/s41598-023-33198-9)
Supplement: Supplementary file 1 — Supplementary Information. [file 41598_2023_33198_MOESM1_ESM.docx]

**Supplementary Table 1.** DSM-5 criteria for conduct disorder and its corresponding questions from Semi-Structured Assessment for the Genetics of Alcoholism (SSAGA) questionnaire acquired by Human Connectome Project (HCP). We identify subjects with prior history of conduct during their childhood (CC) with at least 3 out of 15 DSM-5 conduct symptoms reported present for at least 6 months. “ASP TALLY SHEET PART A” from HCP’s SSAGA was used to identify the presence of the symptoms below for at least 6 months. Note the logical (OR and AND) options applied for each SSAGA questions to fully comply with DSM-5.

|  | DSM-5 Conduct Criteria | SSAGA Question No. of HCP | SSAGA Question |
| --- | --- | --- | --- |
| 1 | Often bullies, threatens, or intimidate others | AS9 | Did people complain that you were often a bully, deliberately hurting, threatening, or being mean to other children? |
| 2 | Often initiates physical fights | AS6  OR  AS6B | Did you 3 or more times start physical fights with your brothers or sisters? |
|  |  |  | Did you 3 or more times start physical fights with persons other than your brothers and sisters? |
| 3 | Has used a weapon that can cause serious physical harm to others (e.g., a bat, brick, broken bottle, knife, gun) | AS20 | Did you ever use a weapon like a stick, gun, or a knife to injure someone (other than in combat or as part of your job)? |
| 4 | has been physically cruel to people | AS19 | (Outside of fighting) have you ever physically injured anyone on purpose? |
| 5 | has been physically cruel to animals | AS10 | Did you ever hurt or injure a pet or any other animal on purpose? |
| 6 | has stolen while confronting a victim (e.g., mugging, purse snatching, extortion, armed robbery) | AS16 | Have you ever taken money or property from someone else by threatening them or using force, like snatching a purse or robbing them? |
| 7 | has forced someone into sexual activity | AS21 | Have you ever forced anyone into any sexual activity? |
| 8 | has deliberately engaged in fire setting with the intention of causing serious damage | AS17 | Did you ever deliberately set fires you were not supposed to? |
| 9 | has deliberately destroyed others’ property (other than by fire setting) | AS18 | Have you ever damaged someone's property on purpose  (other than by fire setting)? |
| 10 | has broken into someone else’s house, building, or car | AS15 | Did you ever break into someone else's home, car, or building (not because you were locked out)? |
| 11 | often lies to obtain goods or favors or to avoid obligations (i.e., “cons” others) | AS11a | Did you often lie to get your own way, or to get out of trouble? |
| 12 | has stolen items of nontrivial value without confronting a victim (e.g., shoplifting, but without breaking and entering; forgery) | AS14  OR  AS14b  OR  AS14d | Did you more than once steal money or things from your family, friends, or relatives? COUNT ONLY IF MORE THAN A FEW DOLLARS. |
|  |  |  | Did you more than once steal or shoplift from stores or from other people? (NO CONFRONTATION) |
|  |  |  | Did you more than once forge anyone's signature on a check or use someone’s credit card without permission? |
| 13 | often stays out at night despite parental prohibitions, beginning before age 13 years | AS4  AND  AS_ao4 | Did you ever stay out late at night without permission, either for 2 or more hours after the curfew your parents set or all night without permission? |
|  |  |  | How old were you the first time? <13 |
| 14 | has run away from home overnight at least twice while living in parental or parental surrogate home (or once without returning for a lengthy period) | AS3  AND  (AS3b  OR  AS3C  OR  AS3C1) | Did you ever run away from home overnight? |
|  |  |  | Did you run away overnight more than once? |
|  |  |  | After you ran away, did you return home? |
|  |  |  | When you ran away, how long did you stay away from home? CHECK TALLY IF AWAY FOR 7 OR MORE DAYS |
| 15 | is often truant from school, beginning before age 13 years | AS1  AND  AS3b | Except for your senior year in high school, did you ever play hooky from school for an entire day? |
|  |  |  | How old were you the first time you played hooky twice in one year? MARK TALLY IF AGE ONSET BEFORE 13. |

**Supplementary Table 2.** All demographics, emotional, personality, and psychiatric evaluations assessed between 40 subjects with prior history of conduct disorder in childhood, and 1166 subjects without history of conduct. All values shown are in mean (SD) format except otherwise reported. *p*-values are derived from parametric or non-parametric statistical tests based on the data distribution for each variable separately. Benjamini-Hochberg’s method was used for multiple comparison correction.

| Variables | HC  n = 1166 | CC  n = 40 | *p* | Corrected *p* |
| --- | --- | --- | --- | --- |
| Demographics |  |  |  |  |
| Age | 28.84 (3.70) | 28.80 (3.38) | 0.914 | 0.914 |
| Sex (M (%)) | 521 (44.7) | 29 (72.5) | 0.001 | 0.008* |
| Years of Education | 14.88 (1.82) | 14.30 (1.86) | 0.046 | 0.118 |
| Income Level | 5.03 (2.17) | 4.38 (2.31) | 0.075 | 0.173 |
| PMAT Correct Responses | 16.68 (4.92) | 15.97 (4.60) | 0.215 | 0.350 |
| Emotion |  |  |  |  |
| Penn Emotion Recognition Test |  |  |  |  |
| Number of Correct Responses | 35.54 (2.56) | 34.85 (3.21) | 0.218 | 0.350 |
| Correct Responses Median Response Time (ms) | 1827.45 (326.16) | 1897.47 (312.05) | 0.119 | 0.236 |
| Number of Correct Anger Identifications | 6.75 (1.03) | 7.10 (0.84) | 0.035 | 0.108 |
| Number of Correct Fear Identifications | 6.90 (1.20) | 6.70 (1.45) | 0.635 | 0.748 |
| Number of Correct Happy Identifications | 7.96 (0.23) | 7.97 (0.16) | 0.652 | 0.751 |
| Number of Correct Neutral Identifications | 7.13 (1.25) | 6.53 (1.71) | 0.007 | 0.037* |
| Number of Correct Sad Identifications | 6.80 (1.14) | 6.55 (1.40) | 0.326 | 0.432 |
| NIH Toolbox: Emotion Battery |  |  |  |  |
| Anger Affect | 47.90 (8.28) | 51.80 (10.92) | 0.009 | 0.040* |
| Anger Aggression | 51.88 (8.69) | 60.47 (10.48) | <0.001 | <0.001* |
| Anger Hostility | 50.65 (8.61) | 51.89 (10.52) | 0.457 | 0.576 |
| Emotional Support | 51.32 (9.57) | 49.86 (12.04) | 0.347 | 0.843 |
| Fear Affect | 50.22 (7.97) | 51.89 (10.92) | 0.263 | 0.377 |
| Fear Somatic Symptoms | 52.02 (8.27) | 52.26 (9.63) | 0.847 | 0.881 |
| Friendship | 50.20 (9.09) | 50.25 (9.97) | 0.773 | 0.836 |
| Instrumental Support | 48.04 (8.95) | 47.49 (12.29) | 0.912 | 0.914 |
| Life Satisfaction | 54.41 (9.19) | 53.21 (10.05) | 0.684 | 0.771 |
| Loneliness | 51.15 (8.77) | 52.97 (9.30) | 0.157 | 0.287 |
| Meaning and Purpose | 51.69 (8.66) | 50.63 (9.38) | 0.462 | 0.576 |
| Perceived Hostility | 48.72 (8.65) | 50.72 (9.67) | 0.252 | 0.372 |
| Perceived Rejection | 48.68 (8.87) | 50.42 (9.67) | 0.175 | 0.299 |
| Perceived Stress | 48.40 (9.11) | 51.00 (10.45) | 0.073 | 0.173 |
| Positive Affect | 50.01 (7.83) | 48.92 (9.23) | 0.315 | 0.429 |
| Sadness | 46.41 (7.97) | 48.67 (8.87) | 0.091 | 0.200 |
| Self-efficacy | 50.80 (8.35) | 51.61 (9.54) | 0.540 | 0.650 |
| Impulsivity (Delay Discounting) |  |  |  |  |
| AUC of trials with 200$ | 0.26 (0.20) | 0.24 (0.23) | 0.278 | 0.388 |
| AUC of trials with 40000$ | 0.51 (0.29) | 0.45 (0.28) | 0.229 | 0.357 |
| Personality |  |  |  |  |
| Agreeableness | 33.44 (5.77) | 30.00 (6.46) | 0.001 | 0.008* |
| Openness | 28.16 (6.27) | 29.45 (6.28) | 0.249 | 0.372 |
| Conscientiousness | 34.47 (5.91) | 33.58 (6.58) | 0.467 | 0.576 |
| Neuroticism | 16.72 (7.34) | 19.15 (8.37) | 0.047 | 0.118 |
| Extraversion | 30.64 (5.96) | 30.75 (6.26) | 0.845 | 0.881 |
| Psychiatry and Life Function |  |  |  |  |
| DSM-Oriented ASR (T scores) |  |  |  |  |
| Depressive Problems | 53.97 (5.71) | 56.23 (8.33) | 0.120 | 0.236 |
| Antisocial Personality Problems | 52.94 (4.53) | 57.73 (9.79) | <0.001 | <0.001* |
| Anxiety Problems | 53.30 (5.19) | 55.52 (7.04) | 0.027 | 0.096 |
| Somatic Problems | 54.07 (5.80) | 56.02 (8.85) | 0.164 | 0.290 |
| Avoidant Personality Problems | 54.40 (6.08) | 56.25 (8.13) | 0.042 | 0.118 |
| Attention-Deficit/Hyperactivity Problems | 54.79 (5.62) | 58.25 (9.40) | 0.010 | 0.040* |
| Syndrome Scale of ASR |  |  |  |  |
| Aggressive Behavior | 52.60 (3.90) | 56.23 (9.31) | 0.002 | 0.015* |
| Rule Breaking Behavior | 53.80 (5.01) | 58.62 (9.36) | <0.001 | 0.001* |
| Intrusive | 53.75 (5.44) | 56.98 (7.40) | 0.002 | 0.012* |
| Anxious/Depressed | 53.97 (6.24) | 55.95 (8.99) | 0.044 | 0.118 |
| Withdrawn | 53.65 (5.75) | 56.38 (9.62) | 0.015 | 0.056 |
| Somatic Complaints | 53.96 (5.91) | 56.35 (9.21) | 0.140 | 0.264 |
| Thought Problems | 53.70 (5.68) | 56.17 (7.67) | 0.010 | 0.040* |
| Attention Problems | 54.90 (5.39) | 57.40 (8.22) | 0.034 | 0.108 |
| Internalizing | 48.60 (10.63) | 52.30 (13.71) | 0.119 | 0.236 |
| Externalizing | 48.70 (8.76) | 55.85 (12.20) | <0.001 | 0.001* |
| ASR Total Score | 47.90 (8.84) | 53.05 (12.04) | 0.003 | 0.016* |
| Significant differences marked with asterisks. HC = Healthy Controls with no History of Conduct, CC = Childhood Conduct History, M = Male, PMAT: Penn Matrix Test of Fluid Intelligence, DSM = The Diagnostic and Statistical Manual of Mental Disorders, ASR = Adult Self-Report. | | | | |

**Supplementary Table 3.** All demographics, emotional, personality, and psychiatric evaluations assessed between two groups in the subjects entering into structural image analysis, after matching based on age and education. All values shown are in mean (SD) format except otherwise reported. Benjamini-Hochberg’s method was used for multiple comparison correction.

| Variables | HC  n = 69 | CC  n = 35 | *p* | Corrected *p* |
| --- | --- | --- | --- | --- |
| Demographics |  |  |  |  |
| Age | 28.90 (3.62) | 28.89 (3.31) | 0.986 |  |
| Sex (M (%)) | 29 (42.0) | 24 (68.6) | 0.019* |  |
| Years of Education | 14.20 (1.97) | 14.40 (1.88) | 0.626 |  |
| Income Level | 4.99 (1.93) | 4.34 (2.44) | 0.148 |  |
| PMAT Correct Responses | 16.20 (4.96) | 16.54 (4.15) | 0.729 |  |
| Emotion |  |  |  |  |
| Penn Emotion Recognition Test |  |  |  |  |
| Number of Correct Responses | 35.72 (2.68) | 34.63 (3.25) | 0.07 | 0.168 |
| Correct Responses Median Response Time (ms) | 1916.25 (516.81) | 1868.97 (310.82) | 0.62 | 0.723 |
| Number of Correct Anger Identifications | 6.83 (1.01) | 7.09 (0.89) | 0.202 | 0.282 |
| Number of Correct Fear Identifications | 6.71 (1.55) | 6.66 (1.35) | 0.864 | 0.864 |
| Number of Correct Happy Identifications | 8.00 (0.00) | 7.97 (0.17) | 0.161 | 0.281 |
| Number of Correct Neutral Identifications | 7.36 (0.91) | 6.51 (1.76) | 0.002 | 0.014* |
| Number of Correct Sad Identifications | 6.83 (0.97) | 6.40 (1.40) | 0.072 | 0.168 |
| NIH Toolbox: Emotion Battery |  |  |  |  |
| Anger Affect | 48.03 (6.96) | 51.64 (10.48) | 0.039 | 0.331 |
| Anger Aggression | 50.01 (8.80) | 60.08 (9.98) | <0.001 | <0.001* |
| Anger Hostility | 50.13 (8.87) | 51.89 (10.57) | 0.373 | 0.649 |
| Emotional Support | 50.03 (10.50) | 49.44 (12.31) | 0.796 | 0.941 |
| Fear Affect | 50.56 (7.27) | 52.77 (10.51) | 0.21 | 0.561 |
| Fear Somatic Symptoms | 50.89 (6.74) | 52.31 (9.49) | 0.382 | 0.649 |
| Friendship | 50.68 (9.31) | 49.45 (9.66) | 0.531 | 0.694 |
| Instrumental Support | 47.37 (9.03) | 47.76 (12.10) | 0.854 | 0.941 |
| Life Satisfaction | 52.83 (9.76) | 54.42 (9.73) | 0.436 | 0.673 |
| Loneliness | 50.14 (9.05) | 51.86 (8.81) | 0.358 | 0.649 |
| Meaning and Purpose | 51.54 (9.66) | 51.75 (9.39) | 0.917 | 0.941 |
| Perceived Hostility | 48.98 (9.13) | 51.61 (9.06) | 0.167 | 0.561 |
| Perceived Rejection | 48.23 (9.61) | 50.65 (9.91) | 0.231 | 0.561 |
| Perceived Stress | 48.62 (9.11) | 51.12 (10.36) | 0.211 | 0.561 |
| Positive Affect | 51.03 (7.99) | 49.83 (9.34) | 0.493 | 0.694 |
| Sadness | 46.12 (7.99) | 49.01 (8.95) | 0.098 | 0.555 |
| Self-efficacy | 51.02 (8.41) | 51.16 (10.00) | 0.941 | 0.941 |
| Impulsivity (Delay Discounting) |  |  |  |  |
| AUC of trials with 200$ | 0.25 (0.18) | 0.24 (0.23) | 0.764 | 0.764 |
| AUC of trials with 40000$ | 0.51 (0.27) | 0.44 (0.27) | 0.204 | 0.408 |
| Personality |  |  |  |  |
| Agreeableness | 34.13 (5.76) | 30.34 (6.68) | 0.003 | 0.015* |
| Openness | 28.22 (6.59) | 28.94 (6.09) | 0.588 | 0.735 |
| Conscientiousness | 34.42 (5.96) | 33.69 (6.50) | 0.566 | 0.735 |
| Neuroticism | 17.00 (7.45) | 18.71 (8.55) | 0.294 | 0.735 |
| Extraversion | 30.64 (6.63) | 31.03 (6.54) | 0.776 | 0.776 |
| Psychiatry and Life Function |  |  |  |  |
| DSM-Oriented ASR (T scores) |  |  |  |  |
| Depressive Problems | 53.88 (5.28) | 55.86 (8.66) | 0.153 | 0.2 |
| Antisocial Personality Problems | 53.03 (4.35) | 57.77 (9.54) | 0.001 | 0.005* |
| Anxiety Problems | 52.84 (4.78) | 55.66 (7.36) | 0.02 | 0.046* |
| Somatic Problems | 54.86 (6.28) | 55.86 (9.20) | 0.515 | 0.515 |
| Avoidant Personality Problems | 54.22 (6.12) | 56.31 (8.38) | 0.149 | 0.2 |
| Attention-Deficit/Hyperactivity Problems | 54.75 (5.15) | 58.74 (9.87) | 0.008 | 0.034* |
| Syndrome Scale of ASR |  |  |  |  |
| Aggressive Behavior | 52.99 (4.34) | 56.11 (9.67) | 0.025 | 0.047* |
| Rule Breaking Behavior | 53.57 (4.41) | 58.89 (9.23) | <0.001 | <0.001* |
| Intrusive | 53.74 (5.35) | 56.91 (7.09) | 0.012 | 0.040* |
| Anxious/Depressed | 53.88 (6.83) | 55.83 (9.25) | 0.228 | 0.258 |
| Withdrawn | 53.03 (4.68) | 56.03 (9.95) | 0.039 | 0.066 |
| Somatic Complaints | 54.59 (6.36) | 56.20 (9.69) | 0.313 | 0.332 |
| Thought Problems | 53.86 (5.81) | 56.14 (8.02) | 0.099 | 0.153 |
| Attention Problems | 54.57 (4.58) | 57.57 (8.64) | 0.022 | 0.046* |
| Internalizing | 48.54 (10.46) | 51.77 (14.11) | 0.19 | 0.230 |
| Externalizing | 49.01 (8.91) | 55.91 (12.27) | 0.001 | 0.005* |
| ASR Total Score | 47.96 (8.82) | 52.89 (12.59) | 0.022 | 0.046* |
| Significant differences marked with asterisks. HC = Healthy Controls with no History of Conduct, CC = Childhood Conduct History, M = Male, PMAT: Penn Matrix Test of Fluid Intelligence, DSM = The Diagnostic and Statistical Manual of Mental Disorders, ASR = Adult Self-Report. | | | | |

**Supplementary Table 4.** Volume measures shown as cubic millimeters (mean (SD)) based on Desikan-Killiany Atlas. *p-*values are for a general linear model to identify differences between groups, with subjects age, sex, and total intracranial volume as covariates. CC = Childhood Conduct history, HC = No Childhood Conduct History.

| Volume | HC  (n = 1166) | CC  (n = 40) | Uncorrected *p* value | Corrected *p* value |
| --- | --- | --- | --- | --- |
| 3^rd^ Ventricle | 767.62 (242.06) | 715.41 (157.14) | 0.017 | 0.249 |
| 4^th^ Ventricle | 1759.28 (597.71) | 1857.76 (758.40) | 0.647 | 0.964 |
| 5^th^ Ventricle | 4.50 (5.96) | 3.38 (4.58) | 0.210 | 0.860 |
| BrainStem | 21835.24 (2481.91) | 22031.95 (2785.48) | 0.592 | 0.964 |
| Anterior Part of Corpus Callusom | 893.77 (143.04) | 894.97 (161.19) | 0.902 | 0.991 |
| Central Part of Corpus Callusom | 500.58 (107.75) | 544.57 (116.90) | 0.020 | 0.249 |
| Midanterior Part of Corpus Callusom | 495.48 (104.25) | 514.16 (105.97) | 0.316 | 0.964 |
| Midposterior Part of Corpus Callusom | 467.06 (99.27) | 476.70 (104.06) | 0.560 | 0.964 |
| Posterior Part of Corpus Callusom | 963.16 (147.74) | 954.86 (171.07) | 0.746 | 0.991 |
| Cerebrospinal fluid | 1061.66 (216.24) | 1072.73 (193.35) | 0.584 | 0.964 |
| Left Accumbens | 563.96 (93.39) | 577.62 (91.51) | 0.911 | 0.991 |
| Left Amygdala | 1553.94 (205.23) | 1605.97 (173.91) | 0.727 | 0.991 |
| Left Caudate | 3808.37 (475.20) | 3827.19 (513.89) | 0.838 | 0.991 |
| Left Cerebellum Cortex | 57026.10 (6087.67) | 57868.32 (6588.41) | 0.645 | 0.964 |
| Left Cerebellum White Matter | 14562.46 (1892.13) | 14956.08 (2693.87) | 0.646 | 0.964 |
| Left Choroid Plexus | 1123.90 (230.70) | 1077.38 (178.08) | 0.027 | 0.262 |
| Left Hippocampus | 4414.71 (483.37) | 4622.97 (528.47) | 0.032 | 0.262 |
| Left Inferior Horn of Lateral Ventricle | 220.46 (134.16) | 199.62 (124.76) | 0.175 | 0.859 |
| Left Lateral Ventricle | 6521.24 (3647.44) | 5212.46 (2039.11) | 0.005 | 0.247 |
| Left Pallidum | 1363.07 (234.88) | 1321.59 (260.73) | 0.066 | 0.400 |
| Left Putamen | 5522.79 (729.43) | 5413.05 (847.78) | 0.051 | 0.358 |
| Left Thalamus | 8449.50 (918.06) | 8713.73 (868.49) | 0.211 | 0.860 |
| Left Ventral Diencephalon | 4195.33 (490.78) | 4264.16 (656.51) | 0.932 | 0.991 |
| Left Vessels | 65.80 (38.63) | 64.19 (33.31) | 0.485 | 0.964 |
| Left White Mater | 220599.87 (27939.02) | 225097.49 (26991.41) | 0.807 | 0.991 |
| Left Cortical Grey Matter | 252078.21 (26850.87) | 258169.11 (23994.20) | 0.529 | 0.964 |
| Optic Chiasm | 230.68 (53.35) | 241.03 (59.41) | 0.900 | 0.991 |
| Right Accumbens | 598.68 (100.14) | 627.89 (90.22) | 0.266 | 0.964 |
| Right Amygdala | 1635.64 (218.37) | 1671.70 (192.87) | 0.884 | 0.991 |
| Right Caudate | 3927.29 (484.53) | 4005.73 (552.62) | 0.552 | 0.964 |
| Right Cerebellum Cortex | 58593.84 (6331.28) | 59851.49 (7381.08) | 0.996 | 0.996 |
| Right Cerebellum White Matter | 14814.42 (2004.09) | 14893.03 (2258.22) | 0.363 | 0.964 |
| Right Choroid Plexus | 1266.34 (287.95) | 1199.70 (178.51) | 0.011 | 0.249 |
| Right Hippocampus | 4483.10 (457.57) | 4600.89 (510.61) | 0.463 | 0.964 |
| Right Inferior Horn of Lateral Ventricle | 235.27 (149.81) | 231.81 (143.39) | 0.493 | 0.964 |
| Right Lateral Ventricle | 6027.38 (3265.96) | 5310.08 (2452.31) | 0.073 | 0.400 |
| Right Pallidum | 1494.45 (198.89) | 1517.19 (245.89) | 0.942 | 0.991 |
| Right Putamen | 5580.52 (642.31) | 5638.03 (522.98) | 0.582 | 0.964 |
| Right Thalamus | 7389.93 (777.70) | 7557.97 (814.13) | 0.523 | 0.964 |
| Right Ventral Diencephalon | 4232.15 (478.80) | 4303.65 (610.14) | 0.985 | 0.996 |
| Right Vessels | 75.04 (37.00) | 78.14 (38.03) | 0.950 | 0.991 |
| Right White Mater | 223777.16 (28349.49) | 227769.81 (26732.30) | 0.633 | 0.964 |
| Right Cortical Grey Matter | 257539.39 (27451.30) | 263544.46 (24801.82) | 0.628 | 0.964 |
| Subcortical Grey Matter | 60899.11 (5454.32) | 61931.38 (5984.86) | 0.872 | 0.991 |
| SupraTentorial Volume | 1031855.11 (112600.69) | 1051386.86 (103995.78) | 0.836 | 0.991 |
| Total White Matter | 444377.02 (56249.72) | 452867.32 (53676.33) | 0.716 | 0.991 |
| Total Grey Matter | 685480.32 (67142.47) | 700628.76 (62955.15) | 0.649 | 0.964 |
| Total Cortical Grey Matter | 509617.61 (54087.06) | 521713.68 (48719.95) | 0.572 | 0.964 |
| White Matter Hypointensities | 852.77 (411.54) | 848.27 (321.48) | 0.623 | 0.964 |

**Supplementary Table 5.** Volumes of hippocampal subfields and amygdala nuclei as cubic millimeters (mean (SD)) compared between two groups using a general linear model with age, gender, education, income level, and total intracranial volume as covariates. *p*-values are all corrected using Benjamini-Hochberg’s FDR method. HC: Healthy Controls, CC: Childhood history of Conduct.

| Volume | HC  (n = 69) | CC  (n = 35) | *p* | Corrected *p* |
| --- | --- | --- | --- | --- |
| Amygdala |  |  |  |  |
| left Lateral nucleus | 654.38 (79.92) | 695.63 (77.06) | 0.121 | 0.266 |
| left Basal nucleus | 451.92 (56.93) | 479.26 (44.49) | 0.064 | 0.186 |
| left Accessory Basal nucleus | 274.31 (35.27) | 290.93 (28.16) | 0.119 | 0.266 |
| left Anterior amygdaloid area | 58.02 (8.16) | 61.33 (7.05) | 0.151 | 0.316 |
| left Central nucleus | 57.09 (9.60) | 58.36 (9.85) | 0.842 | 0.882 |
| left Medial nucleus | 29.84 (6.31) | 30.95 (7.19) | 0.709 | 0.821 |
| left Cortical nucleus | 31.55 (4.63) | 32.87 (4.71) | 0.939 | 0.939 |
| left Corticoamygdaloid transition | 196.91 (26.28) | 211.81 (23.60) | 0.034 | 0.141 |
| left Paralaminar nucleus | 51.58 (6.37) | 54.94 (5.91) | 0.024 | 0.141 |
| left Whole amygdala | 1805.60 (214.27) | 1916.08 (178.72) | 0.062 | 0.186 |
| right Lateral nucleus | 673.14 (78.52) | 721.58 (77.84) | 0.018 | 0.133 |
| right Basal nucleus | 455.62 (57.73) | 476.98 (47.74) | 0.528 | 0.726 |
| right Accessory Basal nucleus | 269.92 (38.06) | 282.16 (28.80) | 0.878 | 0.898 |
| right Anterior amygdaloid area | 62.47 (8.71) | 65.95 (8.26) | 0.338 | 0.533 |
| right Central nucleus | 61.28 (10.83) | 62.78 (11.27) | 0.773 | 0.830 |
| right Medial nucleus | 29.35 (6.60) | 30.44 (5.92) | 0.598 | 0.752 |
| right Cortical nucleus | 28.84 (6.27) | 30.93 (4.23) | 0.640 | 0.782 |
| right Corticoamygdaloid transition | 187.75 (25.18) | 199.46 (22.17) | 0.339 | 0.533 |
| right Paralaminar nucleus | 50.23 (6.16) | 53.04 (5.92) | 0.228 | 0.437 |
| right Whole amygdala | 1818.59 (216.42) | 1923.30 (186.45) | 0.167 | 0.335 |
| Hippocampus |  |  |  |  |
| left CA1 | 677.75 (90.02) | 718.59 (89.78) | 0.083 | 0.214 |
| left CA3 | 207.35 (26.44) | 227.47 (32.27) | 0.002 | 0.036* |
| left CA4 | 248.21 (29.40) | 267.58 (29.03) | 0.003 | 0.036* |
| left fimbria | 97.26 (20.65) | 103.53 (20.18) | 0.304 | 0.533 |
| left GC-ML-DG | 302.63 (36.58) | 326.36 (36.47) | 0.003 | 0.036* |
| left HATA | 61.52 (9.23) | 68.38 (10.07) | 0.003 | 0.036* |
| left hippocampal fissure | 133.03 (24.22) | 140.13 (22.34) | 0.328 | 0.533 |
| left Hippocampal tail | 578.67 (62.03) | 611.16 (61.08) | 0.035 | 0.141 |
| left molecular layer | 408.44 (55.31) | 422.29 (54.23) | 0.435 | 0.627 |
| left parasubiculum | 68.15 (11.67) | 74.05 (15.21) | 0.049 | 0.180 |
| left presubiculum | 304.57 (38.69) | 331.69 (39.92) | 0.005 | 0.045* |
| left subiculum | 455.10 (61.79) | 480.28 (53.12) | 0.079 | 0.214 |
| right CA1 | 716.69 (100.82) | 750.26 (96.82) | 0.239 | 0.438 |
| right CA3 | 228.68 (30.78) | 242.54 (33.41) | 0.055 | 0.186 |
| right CA4 | 260.13 (31.77) | 275.26 (30.09) | 0.035 | 0.141 |
| right fimbria | 90.41 (21.01) | 94.73 (18.93) | 0.737 | 0.821 |
| right GC-ML-DG | 313.32 (37.78) | 332.04 (35.20) | 0.030 | 0.141 |
| right HATA | 61.70 (9.40) | 66.39 (11.05) | 0.119 | 0.266 |
| right hippocampal fissure | 135.72 (35.29) | 137.23 (22.37) | 0.746 | 0.821 |
| right Hippocampal tail | 588.62 (62.44) | 610.80 (63.35) | 0.353 | 0.536 |
| right molecular layer | 415.11 (55.42) | 419.86 (53.28) | 0.712 | 0.821 |
| right parasubiculum | 69.22 (13.14) | 73.48 (16.30) | 0.442 | 0.627 |
| right presubiculum | 290.36 (36.59) | 302.17 (41.81) | 0.597 | 0.752 |
| right subiculum | 441.16 (52.51) | 456.46 (51.63) | 0.554 | 0.738 |
| CA = Cornu Ammonis, GC-ML-DG = Granule Cell and Molecular Layer of Dentate Gyrus, HATA = Hippocampus-Amygdala Transition Area. | | | | |

**Supplementary Table 6.** Results of the interaction analysis including the different behavioral and hippocampal subfields between two groups, as behavioral measures as the outcome, and group X subfield as the predictor variable. Group measure was inserted as a factor: Subjects with prior history of conduct disorder = 1 and unaffected controls = 0.

| Hippocampal Subfield | Behavioral Outcome Measure | Estimate (Std. Error) | *p* | Corrected *p* |
| --- | --- | --- | --- | --- |
| Left Presubiculum | No. of Correct Neutral Identification (Penn Emotion Battery) | 0.002 (0.007) | 0.766 | 0.821 |
|  | Anger Affect Unadjusted (NIH Toolbox) | -0.083 (0.044) | 0.061 | 0.117 |
|  | Anger Aggression Unadjusted (NIH Toolbox) | -0.149 (0.047) | 0.002 | 0.03* |
|  | Agreeableness (Personality) | 0.101 (0.031) | 0.001 | 0.03* |
|  | Antisocial T Score (DSM) | -0.109 (0.034) | 0.002 | 0.03* |
|  | AD/H T Score (DSM) | -0.093 (0.038) | 0.017 | 0.078 |
|  | Aggression T Score (ASR) | -0.145 (0.033) | <0.001 | 0.002* |
|  | Rule Breaking T Score (ASR) | -0.07 (0.035) | 0.048 | 0.106 |
|  | Intrusiveness T Score (ASR) | -0.084 (0.032) | 0.011 | 0.074 |
|  | Total Score (ASR) | -0.13 (0.054) | 0.017 | 0.078 |
|  | Thought Problems T Score (ASR) | 0.012 (0.035) | 0.721 | 0.786 |
|  | Externalizing T Score (ASR) | -0.156 (0.054) | 0.005 | 0.055 |
| Left HATA | No. of Correct Neutral Identification (Penn Emotion Battery) | -0.005 (0.028) | 0.867 | 0.912 |
|  | Anger Affect Unadjusted (NIH Toolbox) | -0.215 (0.177) | 0.227 | 0.297 |
|  | Anger Aggression Unadjusted (NIH Toolbox) | -0.538 (0.192) | 0.006 | 0.056 |
|  | Agreeableness (Personality) | 0.272 (0.127) | 0.035 | 0.093 |
|  | Antisocial T Score (DSM) | -0.316 (0.14) | 0.027 | 0.093 |
|  | AD/H T Score (DSM) | -0.366 (0.153) | 0.019 | 0.081 |
|  | Aggression T Score (ASR) | -0.389 (0.14) | 0.007 | 0.056 |
|  | Rule Breaking T Score (ASR) | -0.292 (0.139) | 0.039 | 0.093 |
|  | Intrusiveness T Score (ASR) | -0.261 (0.132) | 0.051 | 0.109 |
|  | Total Score (ASR) | -0.538 (0.215) | 0.014 | 0.076 |
|  | Thought Problems T Score (ASR) | -0.008 (0.139) | 0.955 | 0.959 |
|  | Externalizing T Score (ASR) | -0.564 (0.218) | 0.011 | 0.074 |
| Left GC-ML-DG | No. of Correct Neutral Identification (Penn Emotion Battery) | -0.011 (0.007) | 0.145 | 0.197 |
|  | Anger Affect Unadjusted (NIH Toolbox) | -0.071 (0.047) | 0.133 | 0.188 |
|  | Anger Aggression Unadjusted (NIH Toolbox) | -0.045 (0.051) | 0.378 | 0.462 |
|  | Agreeableness (Personality) | 0.025 (0.035) | 0.481 | 0.558 |
|  | Antisocial T Score (DSM) | -0.072 (0.038) | 0.059 | 0.117 |
|  | AD/H T Score (DSM) | -0.064 (0.042) | 0.128 | 0.188 |
|  | Aggression T Score (ASR) | -0.085 (0.038) | 0.026 | 0.093 |
|  | Rule Breaking T Score (ASR) | -0.065 (0.037) | 0.085 | 0.141 |
|  | Intrusiveness T Score (ASR) | -0.074 (0.035) | 0.038 | 0.093 |
|  | Total Score (ASR) | -0.118 (0.058) | 0.044 | 0.101 |
|  | Thought Problems T Score (ASR) | 0.002 (0.037) | 0.956 | 0.959 |
|  | Externalizing T Score (ASR) | -0.135 (0.058) | 0.023 | 0.093 |
| Left CA4 | No. of Correct Neutral Identification (Penn Emotion Battery) | -0.014 (0.009) | 0.135 | 0.188 |
|  | Anger Affect Unadjusted (NIH Toolbox) | -0.078 (0.059) | 0.189 | 0.252 |
|  | Anger Aggression Unadjusted (NIH Toolbox) | -0.043 (0.064) | 0.503 | 0.569 |
|  | Agreeableness (Personality) | 0.031 (0.044) | 0.484 | 0.558 |
|  | Antisocial T Score (DSM) | -0.092 (0.047) | 0.053 | 0.11 |
|  | AD/H T Score (DSM) | -0.089 (0.052) | 0.092 | 0.141 |
|  | Aggression T Score (ASR) | -0.103 (0.047) | 0.032 | 0.093 |
|  | Rule Breaking T Score (ASR) | -0.087 (0.047) | 0.066 | 0.117 |
|  | Intrusiveness T Score (ASR) | -0.094 (0.044) | 0.035 | 0.093 |
|  | Total Score (ASR) | -0.135 (0.073) | 0.066 | 0.117 |
|  | Thought Problems T Score (ASR) | -0.002 (0.046) | 0.959 | 0.959 |
|  | Externalizing T Score (ASR) | -0.159 (0.073) | 0.032 | 0.093 |
| Left CA3 | No. of Correct Neutral Identification (Penn Emotion Battery) | -0.015 (0.009) | 0.089 | 0.141 |
|  | Anger Affect Unadjusted (NIH Toolbox) | -0.051 (0.057) | 0.375 | 0.462 |
|  | Anger Aggression Unadjusted (NIH Toolbox) | -0.032 (0.064) | 0.618 | 0.687 |
|  | Agreeableness (Personality) | 0.036 (0.042) | 0.396 | 0.476 |
|  | Antisocial T Score (DSM) | -0.1 (0.045) | 0.029 | 0.093 |
|  | AD/H T Score (DSM) | -0.105 (0.05) | 0.038 | 0.093 |
|  | Aggression T Score (ASR) | -0.086 (0.046) | 0.065 | 0.117 |
|  | Rule Breaking T Score (ASR) | -0.113 (0.044) | 0.013 | 0.076 |
|  | Intrusiveness T Score (ASR) | -0.068 (0.043) | 0.115 | 0.173 |
|  | Total Score (ASR) | -0.121 (0.07) | 0.089 | 0.141 |
|  | Thought Problems T Score (ASR) | -0.046 (0.045) | 0.307 | 0.393 |
|  | Externalizing T Score (ASR) | -0.125 (0.071) | 0.083 | 0.141 |
| CA = Cornu Ammonis, GC-ML-DG = Granule Cell and Molecular Layer of Dentate Gyrus, HATA = Hippocampus-Amygdala Transition Area, AD/H = Attention-Deficit/Hyperactivity, ASR = Adult Self Report. | | | | |
